# Supplementary material for: Characterizing Epitope Binding Regions of Entire Antibody Panels by Combining Experimental and Computational Analysis of Antibody: Antigen Binding Competition
Source: Molecules. 2020 Aug 11;25(16):3659. doi: 10.3390/molecules25163659 (PMC7464469; doi:10.3390/molecules25163659)
Supplement: Supplementary file 1 [file molecules-25-03659-s001.pdf]

molecules

Article

Characterizing Epitope Binding Regions of Entire Antibody Panels by Combining Experimental and Computational Analysis of Antibody: Antigen Binding Competition

Benjamin D. Brooks [1,2,3,*], Adam Closmore [4], Juechen Yang [5], Michael Holland [5], Tina Cairns [3], Gary H. Cohen [3] and Chris Bailey-Kellogg [6]

1 Department of Biomedical Sciences, Rocky Vista University, Ivins, UT 84738, USA
2 Inovan Inc., Fargo, ND 58102, USA
3 Department of Microbiology, School of Dental Medicine, University of Pennsylvania, Philadelphia, PA 19104, USA; tmcairns@upenn.edu (T.C.); ghc@upenn.edu (G.H.C.)
4 Department of Pharmacy, North Dakota State University, Fargo, ND 58102, USA; Adam.Closmore@gmail.com
5 Department of Biomedical Engineering, North Dakota State University, Fargo, ND 58102, USA; juechen.yang@gmail.com (J.Y.); mchollandred@gmail.com (M.H.)
6 Computer Science Department, Dartmouth College, Hanover, NH 03755, USA; cbk@cs.dartmouth.edu
* Correspondence: bbrooks@rvu.edu; Tel.: +1-435-222-1403

Community	gD mAb	Serotype	Neutralizing Activity	Fabs murine/ recombinant	Epitope Residues (experimental)
	E317	Common	Yes	Commercial	38, 132, 139, 140, 215, 222, 235
	DL11	Common	Yes	+/+	38, 132, 140
	MC23	Common	Yes	+/+	213, 216
	MC2	Type-2	Yes	+/+	67, 243-246, 248
	MC5	Common	Yes	+/+	54, 75-77
	MC14	Common	No	+/+	262-272*
	1D3	Common	Yes	+/+	1-29*

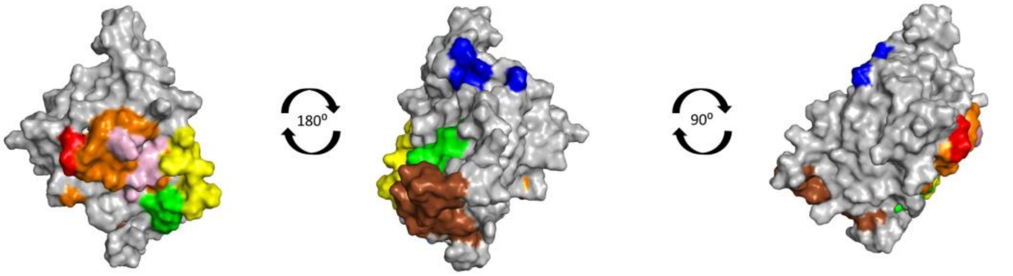

Figure S1. Selected Abs and epitope residues with associated experimental data. Communities are colored on the structure of gD. Orange represents nectin binding region. Face #1 is the nectin binding face. Faces #2-4 are rotated by 90, 180, and 270 degrees around the y-axis. Face #5 is the view from above and Face #6 is the view from below.

Table S1. Agreement between epitope residues targeted by our control antibody, E317, according to the crystal structure vs. those identified from our dock binning based approach.

Crystal Structure	Focused Docking
Gln27	Gln27
Tyr38	Tyr38
Gln132	Gln132
Asp139	Asp139
Ser140	Ser140
Asp215	Asp215
Pro221	Pro221
Arg222	Arg222
Phe223	Phe223
Ile224	
Asn227	
Val231	
Ser235	
Ile238	Ile238
